# Exploratory Cluster-Based Radiographic Phenotyping of Degenerative Cervical Disorder: A Retrospective Study

**DOI:** 10.3390/medicina61050916

**Published:** 2025-05-19

**Authors:** Si-Hyung Lew, Ye-Jin Jeong, Ye-Ri Roh, Dong-Ho Kang

**Affiliations:** 1 College of Medicine, Seoul National University, Seoul 03080, Republic of Korea; 2 ALLIV Healthcare Co., Seoul 05070, Republic of Koreaallivhealthcare@gmail.com (Y.-R.R.); 3 College of Mathematics, Korea University, Seoul 02841, Republic of Korea; 4 College of Nursing, Yonsei University, Seoul 03722, Republic of Korea; 5 Department of Orthopedic Surgery, Samsung Medical Center, Seoul 06351, Republic of Korea

**Keywords:** degenerative cervical disorder, cervical sagittal alignment, clustering analysis, phenotyping, k-means clustering, cervical spine morphology, sagittal vertical axis, cervical lordosis, forward-head posture, long-neck type

## Abstract

*Background and Objectives*: Degenerative cervical myelopathy (DCM), a major subtype of degenerative cervical disorders, presents with diverse sagittal alignment patterns. However, radiography-based phenotyping remains underexplored. This study aimed to identify distinct cervical alignment subgroups using unsupervised clustering analysis and to explore their potential clinical relevance. *Materials and Methods*: We analyzed 1371 lateral cervical radiographs of patients with DCM. C3–C7 sagittal vertical axis (SVA), lordosis, vertical length, and curved length were determined. K-means clustering was applied, and the optimal cluster number was determined using the elbow method and silhouette analysis. Clustering validity was assessed using the Calinski–Harabasz and Davies–Bouldin indices. *Results*: The final clustering solution was validated with a high Calinski–Harabasz index (1171.70) and an acceptable Davies–Bouldin index (0.99) at k = 3, confirming the stability and robustness of the classification. Cluster 1 (forward-head type) exhibited low lordosis (8.3° ± 4.7°), moderate SVA (95.9 ± 60.2 mm), and a compact cervical structure, consistent with kyphotic alignment and forward-head displacement. Cluster 2 (normal) showed the highest lordosis (24.1° ± 6.8°), moderate SVA (70.6 ± 50.2 mm), and balanced sagittal alignment, indicating a biomechanically stable cervical posture. Cluster 3 (long-neck type) displayed the highest SVA (135.6 ± 76.7 mm), the longest vertical and curved lengths, and moderate lordosis, suggesting a structurally elongated cervical spine with anterior head displacement. Significant differences (*p* < 0.01) were observed across all clusters, confirming distinct phenotypic patterns in cervical sagittal alignment. *Conclusions*: This exploratory clustering analysis identified three distinct radiographic phenotypes of DCM, reflecting biomechanical heterogeneity. Although prospective studies linking these phenotypes to clinical outcomes are warranted, our findings provide a framework for personalized spinal care in the future.

## 1. Introduction

Degenerative cervical disorders refer to various conditions that affect the cervical spine, including disk degeneration, spinal stenosis, and cervical spondylosis. Among these, degenerative cervical myelopathy (DCM) specifically involves spinal cord compression, leading to neurological impairment, and is the most common cause of spinal cord dysfunction in adults [1]. Although imaging findings suggestive of cervical myelopathy are occasionally observed in asymptomatic individuals, the clinical presentation of DCM remains highly variable [2]. This heterogeneity complicates prognosis and treatment decision-making, highlighting the need for improved stratification methods.

Recent studies have used machine learning-based cluster analyses to identify distinct DCM phenotypes using patient-reported outcome measures (PROMs) [1,3,4]. A large-scale study that applied unsupervised clustering algorithms identified four clinically distinct DCM phenotypes based on the Nurick score, Neck Disability Index, pain scales, and motor and sensory function scores [4]. These phenotypes exhibit unique symptom distributions and distinct long-term functional trajectories, reinforcing the role of symptom-based classifications in predicting postoperative outcomes [2,3,5]. However, Martin et al. reported that using only PROMs, such as the modified Japanese Orthopedic Association score, has low sensitivity (33%) for detecting symptom progression, suggesting the need for additional clinical data to improve monitoring accuracy [5]. Additionally, a recent systematic review reported that generic PROMs assess overall health but lack sensitivity to disease-specific factors [6]. Therefore, while PROM-based classifications can capture symptom severity, they do not account for non-biomechanical factors such as environmental conditions [7], and biomechanical factors, such as cervical sagittal alignment.

Considering the variability between radiographic findings and clinical presentations, interest in exploring objective imaging-based phenotyping has grown. Photogrammetric tools such as the Postural Assessment Software have been utilized to quantify cervical alignment and postural deviations with high intra- and inter-rater reliability. These tools have shown value in evaluating musculoskeletal dysfunctions and forward-head posture [8,9]. However, these methods primarily rely on external surface landmarks and may not adequately reflect underlying vertebral alignment, especially in deeper spinal structures. Studies have suggested that improper cervical pillow support can worsen cervical alignment and contribute to chronic neck pain [10]. Optimizing sleeping posture to preserve or restore physiological lordosis may theoretically reduce the mechanical stress on the spine. Such biomechanical considerations, which are identifiable through radiographic analysis but not captured by PROMs alone, present potential avenues for developing personalized conservative strategies. In particular, individualized cervical pillows and surgical treatments tailored to specific cervical alignment profiles have shown promising results in preliminary studies [11,12,13,14]. Although these approaches remain exploratory and require further validation, radiography-based phenotypes may help bridge the gap between imaging features and the design of personalized non-surgical and surgical interventions.

To explore these possibilities, we propose an unsupervised clustering analysis using sagittal radiographic parameters extracted from lateral cervical radiographs of patients with DCM. We hypothesized that radiographic alignment patterns could be grouped into distinct cervical phenotypes with biomechanical relevance using unsupervised clustering. In this study, we focus on sagittal plane measurements because sagittal alignment and canal compromise are known to influence clinical outcomes in DCM, whereas coronal plane abnormalities are rare and less associated with neurological impairment [15]. The primary aim of this study was to identify distinct radiographic phenotypes of cervical sagittal alignment in patients with degenerative cervical disorders using unsupervised clustering analysis. The secondary aim was to characterize the biomechanical features of each phenotype and explore their potential clinical relevance.

## 2. Materials and Methods

### 2.1. Study Design and Data Source

This retrospective study analyzed lateral cervical radiograph data obtained from a publicly accessible dataset on the AI Hub, supported by the Ministry of Science and Information and Communications Technology, South Korea (URL: https://aihub.or.kr/aihubdata/data/view.do?currMenu=115&topMenu=100&aihubDataSe=data&dataSetSn=611 (accessed period: 10 February 2025 ~ 11 March 2025). This study was approved by the Institutional Review Board of the Samsung Medical Center (IRB No. 2025-01-017). The retrospective nature of this study and the use of anonymized data waived the requirement for informed consent. The dataset comprised anonymized X-ray images of patients diagnosed with degenerative cervical myelopathy and included pre-labeled cervical lateral X-ray images annotated by medical experts. Degenerative cervical myelopathy was diagnosed by experienced spine specialists from three tertiary medical institutions (Samsung Medical Center, Kyung Hee University Medical Center, and Catholic University Medical Center) based on clinical symptoms, physical examinations, and imaging findings such as cervical radiography, computed tomography, and magnetic resonance image scans. The diagnostic criteria followed the standard clinical guidelines widely accepted in spinal care, including evidence of spinal cord compression, neurological symptoms consistent with cervical myelopathy, and relevant imaging abnormalities. Detailed information on the diagnostic protocols, participating institutions, and labeling processes can be found in the Supplementary Materials. Data were provided through a virtual computing environment for only one month due to data security and privacy issues.

### 2.2. Patient Selection and Annotations

**Figure 1:**
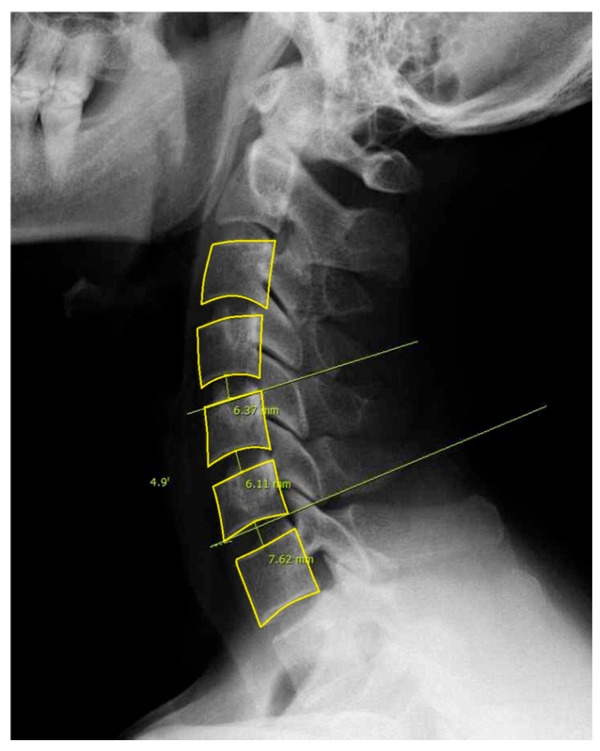
Example of labeled lateral cervical spine radiographs used in this study. The dataset includes segmentation and labeling of the vertebral bodies from C3 to C7.

For this study, we included patients with labeled DCM and utilized pre-labeled cervical lateral radiographs (Figure 1). Specifically, annotations included the following: (1) Identification and marking of anatomical landmarks (key points) on the cervical vertebral bodies (C3–C7). (2) Measurement of cervical sagittal parameters such as cervical lordosis, sagittal vertical axis (SVA), vertical length, and curved length. (3) Calculation and labeling of intervertebral angles and vertebral disk heights. (4) Polygon-based segmentation is used to accurately delineate the cervical vertebral region. All eligible lateral cervical radiographs were included in this study. As unsupervised clustering does not require predefined groupings, we used the full dataset to ensure the robustness and generalizability of the clustering results.

### 2.3. Radiographic Measurements

Owing to the limitations of conducting this study within a virtual computing environment and strictly using the provided dataset, we could not add labeling for C2. Therefore, all radiographic measurements were referred to as C3. A structured processing pipeline was developed to facilitate the automated extraction of sagittal alignment parameters from lateral cervical radiographs. Although this deviates from conventional C2–C7 metrics, we developed a reproducible automated pipeline to ensure internal consistency. Interpretation of the results should take this methodological adjustment into account, particularly when comparing across studies.

Annotation data, originally stored in JSON format, were converted into binary masks to delineate the vertebral structures. The original image dimensions were dynamically retrieved, and annotated regions were transformed into polygon masks, with white-filled areas representing segmented vertebrae. The contours of the vertebrae were then extracted from these masks for subsequent landmark detection.

Key vertebral landmarks, including centroids and endplate points, were identified using the generated masks. The centroid of each vertebra was calculated using the polygon centroid formula to ensure accurate positional representation. For lordosis measurement, the inferior endplate of C3 and superior endplate of C7 were detected by systematically analyzing their respective vertebral contours. Specifically, the inferior endplate of C3 was identified by selecting the lowest boundary point within the leftmost 25% of its contour to ensure a representative point along the anatomical structure. Similarly, the superior endplate of C7 was determined by selecting the highest boundary point within the rightmost 25% of its contour. These boundary points were extracted by sorting the vertebral contour coordinates by the *x*-axis to establish the left and right regions and then identifying the lowest or highest y-coordinates within the designated regions. This structured approach minimized the influence of outlier points and improved measurement reliability. Additionally, the posterior superior aspect of C7 was localized to establish a vertical reference line for sagittal vertical axis (SVA) computation.

Using the extracted vertebral landmarks, four key sagittal alignment parameters were computed: (1) C3 sagittal vertical axis (C3 SVA), defined as the horizontal offset between the centroid of C3 and a vertical plumb line extending from the posterosuperior aspect of C7. This metric quantifies the sagittal alignment deviations. (2) C3-7 lordosis was measured using the Cobb angle calculated between the inferior endplate of C3 and superior endplate of C7. The angle was obtained by identifying the lines tangential to the endplates and computing the angle between them. (3) C3–C7 vertebral body centroid distance, determined as the curved path length connecting the centroids of the C3–C7 vertebral bodies. This was computed by using the sum of the Euclidean distances between consecutive vertebral centroids, approximating the cervical curvature. (4) C3–C7 vertical distance, measured as the absolute vertical displacement between the centroids of C3 and C7, representing the longitudinal span of the cervical segment.

The proposed method ensures consistent and reproducible sagittal alignment measurements by leveraging polygonal segmentation and landmark-based feature extraction. Furthermore, the automated contour analysis minimized the observer variability and enhanced the measurement accuracy. Error-handling mechanisms have been implemented to address missing or ambiguous vertebral structures, improving pipeline robustness.

### 2.4. Phenotypic Classification

Unsupervised machine learning techniques were applied to classify patients into distinct phenotypic subgroups based on the sagittal alignment parameters. The clustering process began with data preprocessing in which all of the radiographic parameters were z-score standardized to ensure comparability.

Next, a principal component analysis (PCA) was performed to reduce dimensionality and enhance visualization. The first two principal components were plotted to assess the separation between the clusters. The number of principal components was determined based on the cumulative explained variance ratio to ensure that the selected components adequately represented the primary variance in the data. Subsequently, K-means clustering was applied to the transformed principal component values, reducing the effects of the curse of dimensionality and facilitating clearer cluster separation in the high-dimensional space.

To determine the optimal number of clusters, the elbow method was applied by evaluating the within-cluster sum of squares and identifying the inflection points on the curve. Additionally, silhouette analysis was conducted to assess cluster cohesion and separation.

Based on these results, the k-means clustering algorithm was implemented using the optimal number of clusters determined by the elbow method and silhouette analysis. To validate the clustering structure, the Calinski–Harabasz and Davies–Bouldin indices were calculated to confirm the robustness of the classification. After clustering, the mean and standard deviation (mean ± SD) of each radiographic parameter within the clusters were computed, and their standardized feature profiles were visualized to highlight distinct sagittal alignment patterns.

### 2.5. Statistical Analysis

Descriptive statistics were used to summarize patient characteristics. Differences between clusters were assessed using one-way analysis of variance (ANOVA) for continuous variables. Post hoc Bonferroni tests were used to identify significant pairwise differences. Statistical significance was set at *p* < 0.05. All statistical analyses were performed using Python (Scikit-learn v1.2.2, SciPy 1.10.1, Statsmodels 0.13.5).

## 3. Results

A total of 1371 patients with degenerative cervical myelopathy were included in the analysis.

### 3.1. Clustering Results

**Figure 2:**
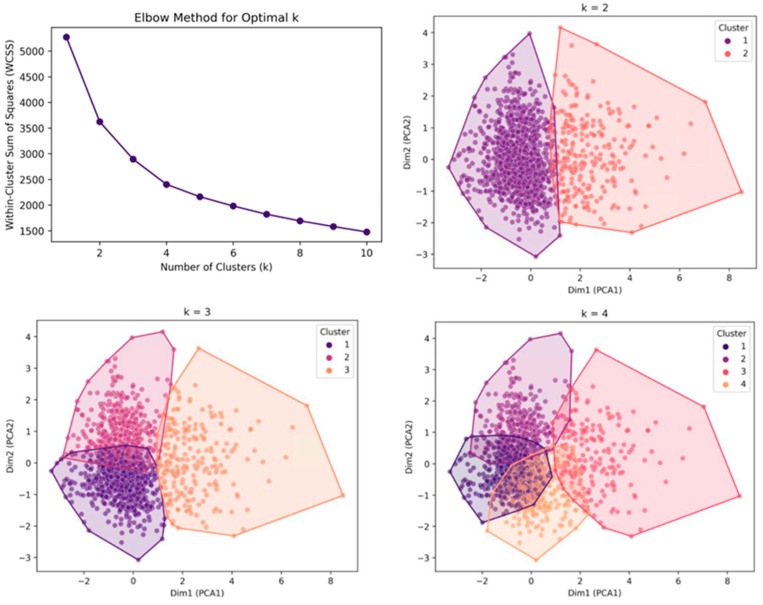
Determination of the optimal number of clusters using the elbow method and silhouette analysis. (Top left) Elbow method using the within-cluster sum of squares for determining the optimal number of clusters (k). (Bottom left and right panels) PCA plots showing clustering results for k = 2, 3, and 4. Each point represents a single case, projected onto the first two principal components (Dim1 and Dim2). Colors indicate cluster assignments. The shaded areas represent the convex hulls of each cluster in PCA space.

Unsupervised k-means clustering was used to classify the patients based on radiographic parameters. The elbow method was used to determine the optimal number of clusters with a visible inflection point between k = 3 and 4 in the within-cluster sum of the squares plot, indicating that both values were potential candidates. However, further evaluation using PCA-based visualizations revealed that the clustering results at k = 4 showed considerable overlap between clusters, leading to poor separation. In contrast, clustering at k = 3 demonstrated better boundary clarity and spatial separation among the groups. Therefore, k = 3 was selected as the most appropriate value (Figure 2).

**Figure 3:**
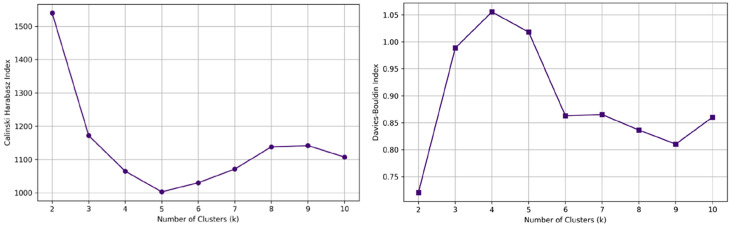
Clustering validation using the Calinski–Harabasz (**left**) and Davies–Bouldin (**right**) indices. The Calinski–Harabasz index decreases sharply up to k = 5 and stabilizes, while the Davies–Bouldin index peaks at k = 4, indicating excessive cluster overlap.

The final clustering solution at k = 3 showed good validity, with a high Calinski–Harabasz index (1171.70) and an acceptable Davies–Bouldin index (0.99), confirming the stability and robustness of the classification (Figure 3).

### 3.2. Cluster Profiles

Table 1 summarizes the distinct sagittal alignment characteristics observed across the three identified phenotypic clusters.

Cluster 3 showed the highest C3–C7 SVA (135.6 ± 76.7 mm), which was significantly greater than Cluster 1 (95.9 ± 60.2 mm, *p* < 0.01) and Cluster 2 (70.6 ± 50.2 mm, *p* < 0.01). This indicates that Cluster 3 had the most anterior cervical alignment. In contrast, Cluster 2 had the lowest SVA, representing a more neutral alignment. The difference between Clusters 1 and 2 was also significant (*p* < 0.01), showing a gradual increase in the SVA from Cluster 2 to Cluster 3.

Regarding C3–C7 lordosis, Cluster 2 showed the greatest C3–C7 lordosis (24.1 ± 6.8°), which was significantly higher than Cluster 1 (8.3 ± 4.7°, *p* < 0.01) and Cluster 3 (13.9 ± 9.4°, *p* < 0.01). Cluster 1 exhibited the lowest lordosis, indicating the highest kyphotic alignment. The difference between Clusters 1 and 3 was also significant (*p* < 0.01).

For C3–C7 length, Cluster 3 demonstrated a markedly longer C3–C7 vertical length (648.0 ± 95.1 mm) than Cluster 1 (457.7 ± 53.8 mm, *p* < 0.01) and Cluster 2 (465.5 ± 56.0 mm, *p* < 0.01). The absence of a significant difference between Clusters 1 and 2 (*p* = 0.079) indicated that vertical elongation was a distinctive feature of Cluster 3. A similar trend was observed in C3–C7 curved length, which was significantly greater in Cluster 3 (841.5 ± 151.3 mm) than in Cluster 1 (550.6 ± 67.0 mm, *p* < 0.01) and Cluster 2 (559.8 ± 74.8 mm, *p* < 0.01). No significant difference was observed between Clusters 1 and 2 (*p* = 0.135).

### 3.3. Formatting of Mathematical Components

**Figure 4:**
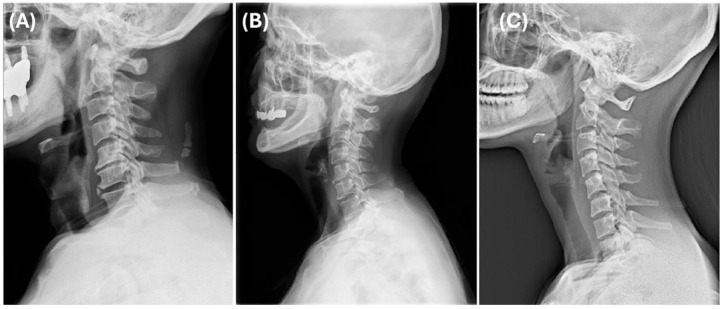
Representative lateral cervical radiographs of each cluster: (**A**) Cluster 1, (**B**) Cluster 2, and (**C**) Cluster 3.

The three identified clusters represented distinct cervical sagittal alignment patterns, each with unique biomechanical characteristics. Cluster 1 showed the lowest degree of lordosis and a moderate SVA, consistent with kyphotic alignment and anterior head displacement (Figure 4A). Cluster 2 exhibited the greatest lordosis and a well-balanced cervical posture (Figure 4B). Cluster 3 was characterized by the most anteriorly displaced cervical alignment (highest SVA) along with the longest vertical and curved lengths, indicating an elongated cervical spine morphology (Figure 4C).

**Figure 5:**
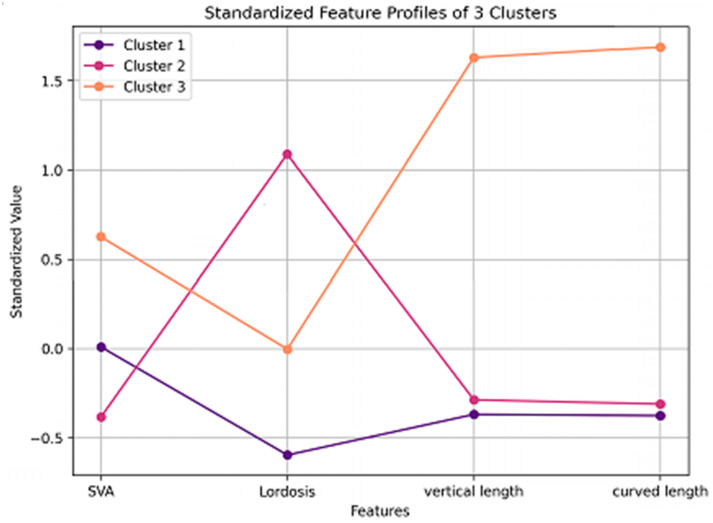
Z-score standardized profiles of the sagittal alignment parameters across the three identified clusters.

The standardized feature profile analysis (Figure 5) further supported these classifications, highlighting distinct sagittal alignment variations. Cluster 1 (forward-head type) demonstrated reduced lordosis and moderate SVA, suggesting a kyphotic cervical curve and anterior head posture. Cluster 2 (normal) showed the highest lordosis. Cluster 3 (long-neck type) exhibited the highest SVA and the longest cervical spine.

## 4. Discussion

We identified three distinct phenotypic subgroups of degenerative cervical myelopathy based on the sagittal alignment parameters. The forward-head type exhibited reduced lordosis and moderate sagittal vertical axis, indicating kyphotic cervical alignment and anterior head displacement. The normal type had the highest lordosis and balanced sagittal alignment, indicating a stable cervical posture. The long-neck type demonstrated the highest sagittal vertical axis and the longest cervical spine dimensions, suggesting a structurally elongated cervical morphology. These phenotypic classifications suggest biomechanical differences that warrant further investigation regarding their potential relevance in degenerative cervical myelopathy management.

Cluster 1 was classified as the forward-head type, showing the lowest degree of lordosis and a moderate SVA, consistent with kyphotic alignment and anterior head displacement (Figure 4A). Cluster 2 was considered the normal type, exhibiting the greatest lordosis and a well-balanced cervical posture (Figure 4B). Cluster 3 was defined as the long-neck type, characterized by the most anteriorly displaced cervical alignment (highest SVA) along with the longest vertical and curved lengths, indicating an elongated cervical spine morphology (Figure 4C).

Previous phenotyping studies on DCM have mainly used PROMs for classification. Khan et al. applied machine learning algorithms to predict postoperative health-related quality of life [1], whereas Badhiwala et al. explored how different phenotypes relate to surgical outcomes [2]. Recently, Pedro et al. identified four distinct DCM phenotypes using cluster analysis, each with unique clinical characteristics and long-term neurological outcomes [4]. These findings support the potential of phenotyping to improve our understanding of DCM pathophysiology and promote more personalized treatment strategies, which may be difficult to achieve using symptom-based methods alone. However, because these studies relied on subjective symptom reports, their findings may have been affected by non-biomechanical factors. In contrast, our radiographic-based phenotyping provides an objective classification that reflects the underlying structural and biomechanical differences, thus complementing existing approaches.

The choice of surgical approach for DCM varies according to the cervical alignment type. This exploratory analysis provides structural observations that can support future objective classifications. Identifying forward-head, normal, and long-neck phenotypes may help generate hypotheses for tailoring surgical strategies for specific sagittal alignment types. The forward-head type (Cluster 1), characterized by reduced lordosis and kyphotic alignment, shares biomechanical similarities with K-line-negative DCM patients [16]. The K-line is defined as the line connecting the midpoints of the spinal canal at C2 and C7 on lateral radiographs. Patients classified as K-line-negative have pathologies such as ossification of the posterior longitudinal ligament (OPLL) extending posteriorly to this line, indicating a need for more complex or combined surgical approaches to achieve adequate spinal cord decompression and stability [13]. Several studies have reported that K-line-negative patients have poor neurological recovery after laminoplasty due to restricted spinal cord mobility [16,17]. The long-neck type (Cluster 3), with its elongated cervical structure and highest SVA, shares biomechanical similarities with patients with increased K-line tilt in the DCM [16,18]. The K-line tilt, defined as the angle between the K-line and the vertical line, is positively correlated with the C2–C7 SVA [16]. Studies have shown that a K-line tilt > 10° independently predicts poor patient outcomes and postoperative kyphosis after laminoplasty in multilevel DCM [19,20]. Furthermore, Sakai et al. recently reported that a K-line tilt >20° increased the risk of kyphotic deformity even in patients with K-line positive cervical OPLL, making laminoplasty alone insufficient in such cases [21]. Therefore, our identification of these two phenotypes raises the hypothesis that surgical strategies targeting anterior compressive lesions may be beneficial, given their higher risk of postoperative kyphosis; however, this hypothesis requires further validation. Further studies are needed to confirm whether alignment-based classifications can be directly applied in surgical decision-making. The normal type (Cluster 2) represents a biomechanically optimal alignment pattern and may serve as a therapeutic target for interventions aimed at restoring cervical alignment in other phenotypes.

The identification of distinct cervical alignment phenotypes raises the possibility of informing the future development of customized conservative treatments, particularly cervical pillows, which are widely used in the management of neurological symptoms and axial neck pain in DCM. Haoxin et al. reported that a 3D-printed, patient-specific cervical correction pillow effectively restored cervical lordosis with 88% clinical improvement after 24 weeks, reduced neck and shoulder pain, and improved sleep quality [11]. Türkmen et al. reported that the effects of pillow materials on comfort and spinal support vary depending on spinal alignment, such as forward-head posture [12]. Lavin et al. demonstrated that semi-customized cervical pillows that provided firm lordotic curvature support effectively managed neck pain [22]. Yamada et al. indicated that careful pillow height adjustments significantly improved neck pain and related physical symptoms [10]. Current recommendations typically overlook the variations in individual cervical alignments. Our findings may inform the design of future customized pillow strategies, although further clinical validation is required. It is important to note that in patients with DCM, symptoms may arise from the spinal cord compression itself, whereas others may experience mechanical axial neck pain not directly related to cord compression. Customized pillows based on cervical alignment phenotypes may be more applicable to mechanical neck pain than to myelopathy-related deficits. Additionally, in patients with cervical cord compression or neurological symptoms that worsen with flexion or extension postures compared to neutral postures, adjusting the sleeping cervical curvature and height to reduce compression may help minimize cord compression during sleep and alleviate sleep-related cervical discomfort. Therefore, designing a pillow support to maintain a spinal position that avoids pathological flexion or extension might provide therapeutic benefits for select patients with cervical myelopathy. Regarding mechanical neck pain, forward-head type patients may benefit from pillows with suitable heights to restore lordosis, prevent excessive lordotic curvature, and minimize discomfort. Normal type patients require pillows that preserve their naturally balanced cervical alignment. For long-neck type patients (Cluster 3), pillows should offer extended curve lengths and structural support to maintain cervical lordosis. Of the three phenotypes, the long-neck type patients had the longest cervical alignment, with mean C3–C7 vertical and curved lengths of 648.0 mm and 841.5 mm, respectively. Pillows for this group should include an extended neck support that matches these dimensions. In addition, since this group also demonstrated the highest SVA of 135.6 mm, the occipital portion of the pillow may need to be elevated higher than that of other clusters to provide appropriate support based on each individual’s SVA. Although further studies are needed to determine whether personalized pillows could affect symptoms or functional outcomes in DCM, our findings provide preliminary structural insights that could contribute to future strategies targeting biomechanical factors associated with axial neck pain. Surface-based photogrammetric tools, which enable the non-invasive assessment of cervical alignment in functional and sleep-related postures, may complement radiographic phenotyping and support the development of personalized pillow designs tailored to each alignment type.

Nevertheless, it is important to emphasize that our results are preliminary and hypothesis-generating. Based on this observational analysis, we cannot conclude causation or recommend changes in clinical practice. While these radiographic phenotypes may hypothetically support personalized treatment strategies, our findings do not justify any changes in current practice. The association between these phenotypes and clinical outcomes requires confirmation in future prospective studies to determine their prognostic or therapeutic significance.

This study has several limitations. Firstly, the cross-sectional nature of this study limited the ability to determine whether the identified radiographic phenotypes are predictive of clinical outcomes. Longitudinal studies are required to examine how these phenotypes relate to disease progression and treatment outcomes over time. Secondly, because the dataset did not include labeled C2 and considerable time was consumed in preparing the virtual environment and installing the necessary packages after approval, the actual analysis time was reduced to approximately two weeks. Consequently, all measurements used C3 as the reference, potentially affecting clinical applicability and generalizability. Future research using C2 lordosis and C2 SVA could better evaluate clinical relevance. Thirdly, although the annotation quality was indirectly supported by the reported performance metrics of the AI model used by the dataset provider—specifically, a correlation coefficient of 0.71 for intervertebral disk height measurement, which is relevant to our study—we were unable to independently verify the accuracy of landmark labeling due to the limited access period and the absence of raw images or manual labeling tools. Future studies should aim to directly validate landmark accuracy and ensure inter-rater reliability by using datasets with full access to annotation procedures or by implementing independent manual labeling protocols. Fourth, this study did not include clinical outcome data; therefore, we could not assess whether the identified radiographic phenotypes had a prognostic value. Future studies that combine radiographic and clinical datasets are required to evaluate their roles in treatment planning and outcome prediction. Fifth, the use of a dataset composed exclusively of Korean patients may limit the generalizability of our findings to other populations. Finally, although PCA was primarily used to visualize cluster separation, the selection of principal components was based on the cumulative explained variance. Two components were selected to sufficiently capture key variations in the data. However, the PCA-transformed features were not used as direct inputs for clustering. Future studies should evaluate the potential benefits of integrating PCA into the clustering process.

## 5. Conclusions

This exploratory study identified three distinct radiographic phenotypes of degenerative cervical myelopathy based on sagittal alignment: forward-head, normal, and long-neck type. These phenotypes exhibited consistent differences in cervical alignment parameters, suggesting underlying biomechanical diversity among patients. However, owing to the cross-sectional and observational nature of this study, these findings are hypothesis-generating and should not be directly extrapolated to clinical practice. Further prospective studies integrating longitudinal clinical outcomes are necessary to validate the prognostic and therapeutic significance of these radiographic phenotypes. In the future, radiography-based phenotyping may complement patient-reported outcomes and contribute to a more comprehensive approach to personalized spinal care, pending further validation.

## Figures and Tables

**Table 1:** Comparison of radiographic parameters across phenotypic clusters.

Parameters	Cluster 1 (Mean ± SD) (N = 703)	Cluster 2 (Mean ± SD) (N = 387)	Cluster 3 (Mean ± SD) (N = 228)	ANOVA		Post Hoc Pairwise Comparison		
				F Statistic	*p*	1 vs. 2	1 vs. 3	2 vs. 3
C3–C7 SVA (mm)	95.9 ± 60.2	70.6 ± 50.2	135.6 ± 76.7	82.4	<0.01	<0.01	<0.01	<0.01
C3–C7 Lordosis (°)	8.3 ± 4.7	24.1 ± 6.8	13.9 ± 9.4	755.5	<0.01	<0.01	<0.01	<0.01
C3–C7 Vertical Length (mm)	457.7 ± 53.8	465.5 ± 56.0	648.0 ± 95.1	825.0	<0.01	0.08	<0.01	<0.01
C3–C7 Curved Length (mm)	550.6 ± 67.0	559.8 ± 74.8	841.5 ± 151.3	977.9	<0.01	0.14	<0.01	<0.01

SD, standard deviation; SVA, sagittal vertical axis.

## Data Availability

The data used in this study were obtained from publicly available sources provided by the Ministry of Science and ICT, South Korea, via AI Hub (URL: https://aihub.or.kr/ (accessed period: 10 February 2025 ~ 11 March 2025)). The dataset consists of anonymized cervical lateral X-ray images and related metadata. Access to this dataset is subject to the policies and regulations of the AI Hub. The authors do not have the authority to distribute the data directly. Researchers interested in accessing the dataset should refer to the official AI Hub platform for further details on data availability and access permissions.

## Supplementary Materials

The following supporting information can be downloaded at: https://www.mdpi.com/article/10.3390/medicina61050916/s1.

## Abbreviations

The following abbreviations are used in this manuscript:

DCD	Degenerative cervical disorder
DCM	Degenerative cervical myelopathy
PROMs	Patient-reported outcome measures
SVA	Sagittal vertical axis
PCA	Principal component analysis
TOS	Thoracic outlet syndrome
DSS	Droopy shoulder syndrome
ANOVA	Analysis of variance
SD	Standard deviation
