# Supplementary material for: Exploratory Cluster-Based Radiographic Phenotyping of Degenerative Cervical Disorder: A Retrospective Study
Source: Medicina (Kaunas). 2025 May 19;61(5):916. doi: 10.3390/medicina61050916 (PMC12112840; doi:10.3390/medicina61050916)
Supplement: Supplementary file 1 [file medicina-61-00916-s001.zip › medicina-3627471-supplementary.pdf]

<table>
<tr><th>Field</th><th>Data Type</th><th>Data Volume</th><th>Original Data Format[2]</th><th>Labeling Format[3]</th><th>Labeling Type[4]</th></tr>
<tr><td>Healthcare</td><td>Image</td><td>1,393,475</td><td>Dicom</td><td>json</td><td>Segmentation (Image)</td></tr>
<tr><th>Data Source[5]</th><th>Data Year</th><th>Building Institution (Lead)</th><th>Processing Institution</th><th>Verification Institution</th><th></th></tr>
<tr><td>Self-Collected</td><td>2021</td><td>Catholic University Industry-Academic Cooperation Group</td><td>Miso Information Technology Co., Ltd.</td><td>Innerwave Co., Ltd.</td><td></td></tr>
<tr><th rowspan="2">Data Inquiry Contact</th><th>Organization</th><th>Contact Person</th><th>Phone Number (Landline preferred)</th><th>Email Address</th><th></th></tr>
<tr><td>Catholic University Industry-Academic Cooperation Group</td><td>Jin-sung Kim</td><td>02-2258-6844</td><td>mdl1david@catholic.ac.kr</td><td></td></tr>
<tr><th>Data Description</th><td colspan="5">70. Multimodality data for diagnosis and treatment of degenerative spinal diseases</td></tr>
<tr><th>Keywords</th><td colspan="5">Degenerative Cervical Spinal Stenosis, Thoracic Spine, Degenerative Lumbar Disc Disease, Degenerative Lumbar Spinal Stenosis</td></tr>
</table>

Data Description

This dataset comprises multimodal image data aimed at diagnosing and treating degenerative spinal diseases. Specifically, it addresses four major degenerative spinal conditions:

1. Degenerative Cervical Spinal Stenosis (DCS)
2. Thoracic Myelopathy (DTM)
3. Degenerative Lumbar Disc Disease (DLD)
4. Degenerative Lumbar Spinal Stenosis (DLS)

Objectives

To acquire large-scale normal and pathological data of degenerative spinal diseases (including disc disorders and stenosis) involving cervical, thoracic, and lumbar regions.

To analyze correlations between radiological diagnoses (image-based) and clinical symptoms using artificial intelligence (AI).

To develop evidence-based medical datasets to enhance prevention and treatment strategies for high-frequency spinal diseases.

To efficiently and accurately predict disease progression and select appropriate treatment methods using multimodal imaging, including MRI, particularly for moderate to severe degenerative spinal conditions.

To support AI-based clinical decision-making by establishing multicenter clinical datasets, correlating radiological diagnoses with clinical presentations, and facilitating optimal treatment selection.

Key Application Areas

Provision of AI-based healthcare services for degenerative spinal diseases.

Development of profitable service models based on data.

Establishment of an AI ecosystem related to healthcare services.

Data Composition

Data sourced from eight medical institutions, comprising over 1,393,475 annotated images.

De-identified data ensuring patient privacy protection.

Includes over 3,000 patients and more than 10,000 image cases.

Data includes X-ray, MRI, and CT images across the specified degenerative spinal diseases.

Labeling Details

Detailed segmentation labeling on spinal images to facilitate precise identification of pathological regions, disc height and angles, and spinal cord area measurements.

Labeling schema provided in JSON format to enable integration into AI modeling processes.

Clinical and Technical Uses

Measurement algorithms for disc height and angles using segmented spinal vertebral regions from lumbar X-ray images.

AI models for automatic detection of posterior longitudinal ligament ossification (OPLL) using cervical CT images.

Segmentation and area measurement models for thoracic spine regions using T2 axial plane MRI data.

Category Definition

The data category definitions are submitted in an Excel file attached separately, following the provided example format.

Data	Cervical X-ray	Thoracic MRI	Thoracic CT
example	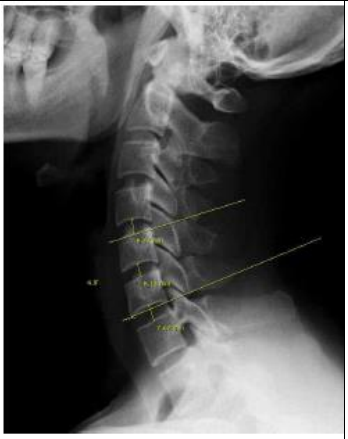 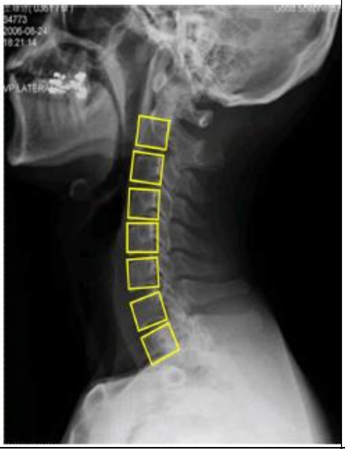	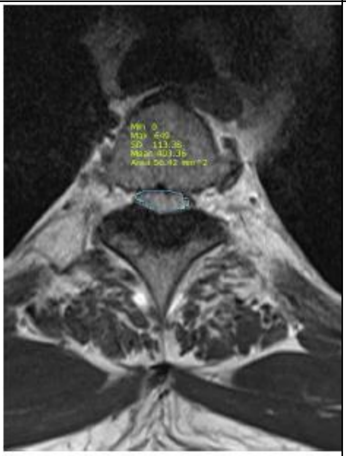 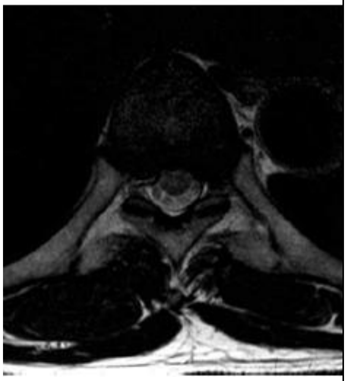	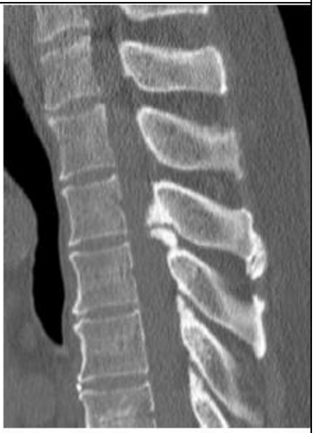 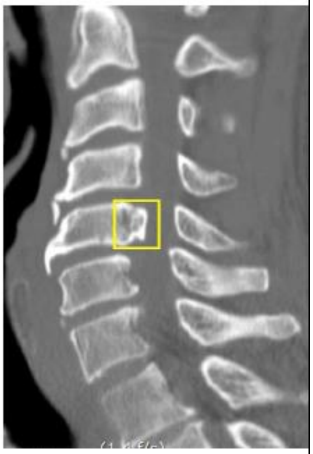
Data composition	Height of intervertebral disc<br>Bounding box of vertebral body	Area of T5-6 in T2 axial image<br>Outline of spinal canal in T2 axial image	Thoracic OPLL evaluation<br>Bounding box of thoracic OPLL
Data format	Original: dicom<br>Learning data: jpg<br>Annotation: txt<br>ROI: JSON	Original: dicom<br>Learning data: jpg<br>Annotation: txt<br>ROI: NIfTI	Original: dicom<br>Learning data: jpg<br>Annotation: txt<br>ROI: JSON

Example of labeling according to specific disease

구분	X-ray	MRI	CT
Degenerative cervical stenosis	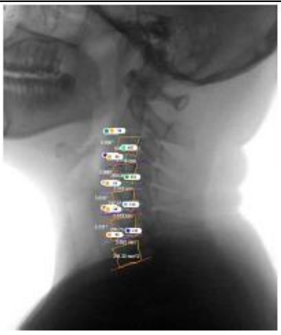	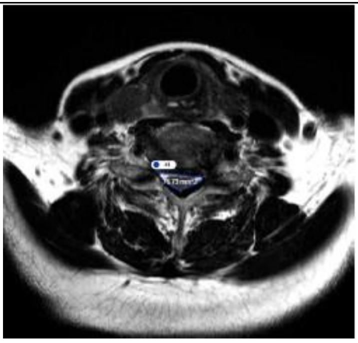	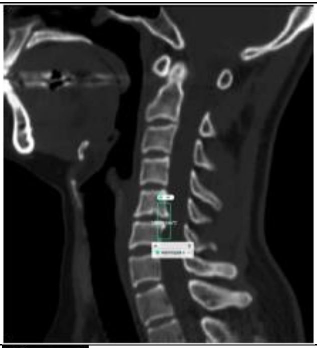
Thoracic myelopathy		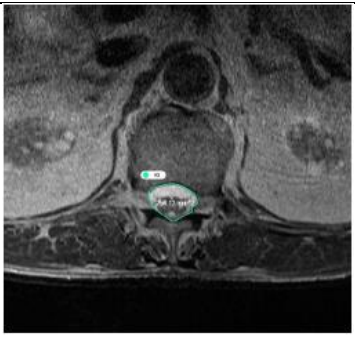	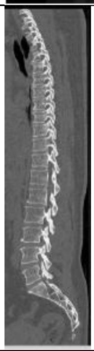
Degenerative lumbar disc disease	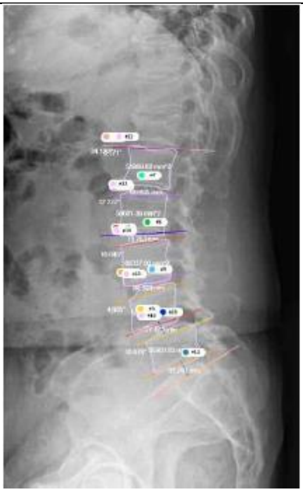	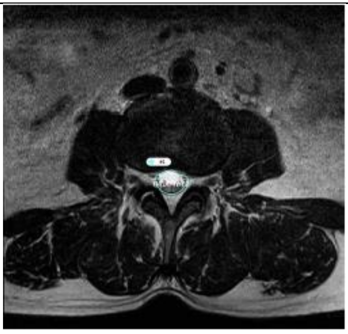	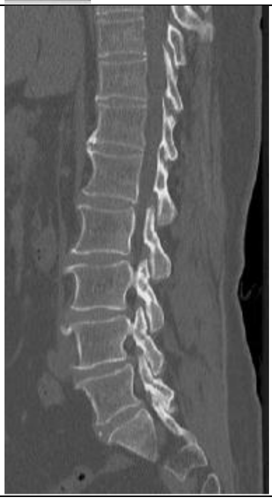

Degenerative lumbar stenosis	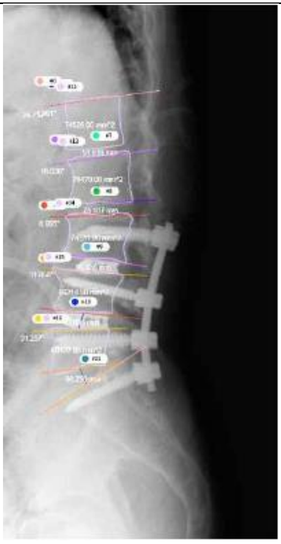	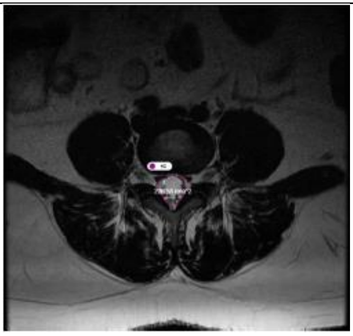	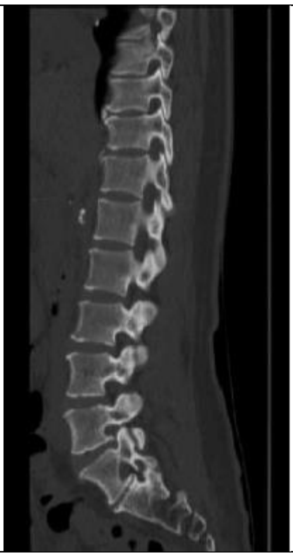
